# Supplementary material for: Resolving the full spectrum of human genome variation using Linked-Reads
Source: Genome Res. 2019 Apr;29(4):635–45. doi: 10.1101/gr.234443.118 (PMC6442396; doi:10.1101/gr.234443.118)
Supplement: Supplemental Material [file supp_gr.234443.118_Supplemental_longranger-2.2.zip › longranger-2.2.tar.bz2!/lr2.2-public/lib/stan/cmdstan/stan/doc/api/html/distance_8hpp_source.html]

Stan: src/stan/math/matrix/distance.hpp Source File


|  |  |
| --- | --- |
|  | Stan  2.5.0  probability, sampling & optimization |


- Main Page
- Related Pages
- Namespaces
- Classes
- Files

- File List
- File Members

All Classes Namespaces Files Functions Variables Typedefs Enumerations Enumerator Friends Macros Pages

- src
- stan
- math
- matrix

distance.hpp

Go to the documentation of this file.

1 #ifndef STAN\_\_MATH\_\_MATRIX\_\_DISTANCE\_HPP

2 #define STAN\_\_MATH\_\_MATRIX\_\_DISTANCE\_HPP

3

4 #include <stan/math/matrix/Eigen.hpp>

5 #include <stan/meta/traits.hpp>

6 #include <boost/math/tools/promotion.hpp>

7 #include <stan/math/matrix/squared\_distance.hpp>

8 #include <stan/math/error\_handling/matrix/check\_vector.hpp>

9 #include <stan/math/error\_handling/matrix/check\_matching\_sizes.hpp>

10

11 namespace stan {

12  namespace math {

13

23  template<typename T1, int R1,int C1, typename T2, int R2, int C2>

24  inline typename boost::math::tools::promote\_args<T1,T2>::type

25  distance(const Eigen::Matrix<T1, R1, C1>& v1,

26  const Eigen::Matrix<T2, R2, C2>& v2) {

27  using std::sqrt;

28  stan::math::check\_vector("distance(%1%)",v1,"v1",(double\*)0);

29  stan::math::check\_vector("distance(%1%)",v2,"v2",(double\*)0);

30  stan::math::check\_matching\_sizes("distance(%1%)",v1,"v1",

31  v2,"v2",(double\*)0);

32  return sqrt(squared\_distance(v1,v2));

33  }

34  }

35 }

36 #endif

stan::math::check\_vector

bool check\_vector(const char \*function, const Eigen::Matrix< T, R, C > &x, const char \*name, T\_result \*result)

**Definition:** check\_vector.hpp:14

check\_matching\_sizes.hpp

traits.hpp

stan::agrad::sqrt

fvar< T > sqrt(const fvar< T > &x)

**Definition:** sqrt.hpp:15

Eigen.hpp

check\_vector.hpp

stan::math::check\_matching\_sizes

bool check\_matching\_sizes(const char \*function, const T\_y1 &y1, const char \*name1, const T\_y2 &y2, const char \*name2, T\_result \*result)

**Definition:** check\_matching\_sizes.hpp:15

squared\_distance.hpp

stan::math::squared\_distance

boost::math::tools::promote\_args< T1, T2 >::type squared\_distance(const Eigen::Matrix< T1, R1, C1 > &v1, const Eigen::Matrix< T2, R2, C2 > &v2)

Returns the squared distance between the specified vectors.

**Definition:** squared\_distance.hpp:22

stan::math::distance

boost::math::tools::promote\_args< T1, T2 >::type distance(const Eigen::Matrix< T1, R1, C1 > &v1, const Eigen::Matrix< T2, R2, C2 > &v2)

Returns the distance between the specified vectors.

**Definition:** distance.hpp:25


---

[ Stan Home Page ]

*© 2011–2014,
Stan Development Team.*
